# Does prophylactic inferior vena cava filter reduce the hazard of pulmonary embolism and mortality in severe trauma? A single center retrospective comparative study

**DOI:** 10.1016/j.ejro.2020.100299

**Published:** 2020-12-10

**Authors:** Thien Trung Tran, Haraldur Bjarnason, Jennifer McDonald, Brian Goss, Brian Kim, Damon E. Houghton, Knut Stavem, Nils Einar Kløw

**Affiliations:** a Institute of Clinical Medicine, Faculty of Medicine, University of Oslo, Norway; b Department of Diagnostic Imaging and Intervention, Akershus University Hospital, Lørenskog, Norway; c Department of Radiology, Mayo Clinic, Rochester, MN, USA; d Department of Radiology, Kingman Regional Medical Center, AZ, USA; e Division of Trauma, Critical Care and General Surgery, Mayo Clinic, Rochester, MN, USA; f Department of Hematology, Mayo Clinic, Rochester, MN, USA; g Department of Pulmonary Medicine, Akershus University Hospital, Lørenskog, Norway; h Division of Radiology and Nuclear Medicine, Oslo University Hospital Ullevål, Oslo, Norway

**Keywords:** IVC, Inferior Vena Cava, VTE, venous thromboembolism, PE, pulmonary embolism, DVT, deep venous thrombosis, ISS, Injury Severity Score, AIS, Abbreviated Injury Scale, GCS, Glasgow Coma Scale, HR, Hazard Ratio, Multiple trauma, Critical care, Pulmonary embolism, Venous thromboembolism, Inferior vena cava filter, Patient outcome

## Abstract

**Objectives:**

Use of inferior vena cava (IVC) filters in patients following severe trauma without recent history of venous thromboembolism (VTE) is controversial. Our objective was to determine if IVC filter placement in the setting of severe trauma effects the hazard of in-hospital pulmonary embolism (PE), deep venous thrombosis (DVT) and mortality.

**Methods:**

This retrospective study recruited patients from a single Level I Trauma Center between 1/2008 and 12/2013. Inclusion criteria were age>15 years, Injury Severity Score (ISS)>15 and survival>24 h after hospital admission. Patients with VTE diagnosed prior to IVC filter placement were excluded. A Cox proportional hazards regression model was used, adjusting for immortal time bias with landmark analysis at predefined time after injury. Differences between IVC filter and non-IVC filter groups were adjusted using propensity score.

**Results:**

In total 1451 patients were reviewed; 282 patients received an IVC filter and 1169 patients had no IVC filter placed. The mean age was 45.9 vs. 56.9 years and the mean ISS was 29.8 vs. 22.6 in the IVC filter and the non-IVC filter group, respectively. IVC filter placement was not associated with the hazard of PE (HR = 0.46; 95 % CI, 0.12,1.70; P = 0.24) or mortality (HR = 1.02; 95 % CI 0.60,1.75; P = 0.93). However, IVC filter placement was associated with the hazard of DVT (HR = 2.73; 95 % CI, 1.28,5.85; P = 0.01).

**Conclusions:**

In patients with severe trauma, those with prophylactic IVC filter placement did not have a reduced hazard of PE or mortality, but an increased hazard of DVT was observed.

## Highlights

• Severely injured trauma patients are at high risk of venous thromboembolism.
• Use of IVC filters in trauma patients without recent history of venous thromboembolism is controversial.
• IVC filter placement did not reduce the hazard of pulmonary embolism or mortality but may pose an increased hazard of deep venous thrombosis.

## 1 Introduction

Trauma patients have increased risk of venous thromboembolism (VTE), which comprises deep venous thrombosis (DVT) and pulmonary embolism (PE). Estimates of VTE risk vary widely in this population, ranging from 12 % to 65 % in patients who do not receive pharmacological thromboprophylaxis (low-molecular-weight-heparin or unfractionated-heparin) [1]. The increased risk of VTE is due to venous stasis, intimal injury, and increased coagulability according to Virchow’s triad. In severe trauma, thromboembolic complications may coexist along with uncontrolled hemorrhage and organ failure in a complex condition termed trauma-induced coagulopathy [2].

Use of inferior vena cava (IVC) filters in patients after severe trauma without recent history of VTE is relatively common but controversial. According to the Society of Interventional Radiology (SIR), IVC filters are typically placed in three clinical scenarios: (1) in patients with VTE and classic indications; (2) in patients with VTE and extended indications; and (3) in patients without VTE for primary prophylaxis against PE [3].

Observational studies have reported lower risk of PE and PE-related death in trauma patients with IVC filters, however the levels of evidence are low [4]. Many of these studies may suffer from selection bias and immortal time bias. Selection bias may be present if IVC filter placement were preferentially prescribed to patients based on their underlying risk profile [5]. Immortal time bias may be present if an outcome such as mortality cannot occur during a period of cohort follow-up [6]. The person-time between injury and IVC filter placement is considered immortal and does not exist in the non-IVC filter cohort. These biases may account for the differences seen between observation trials and a recent randomized trial that did not show a benefit of early prophylactic placement of IVC filter in reducing the incidence of symptomatic PE or mortality [7].

In this retrospective study, we examined whether IVC filter placement reduced the hazard of in-hospital PE and mortality when accounting for immortal time bias and selection bias with detailed trauma scores in a propensity score adjustment.

## 2 Materials and methods

### 2.1 Study design and setting

This was an observational study of trauma patients admitted to one trauma center and recorded prospectively in a trauma registry. Mayo Clinic is an American College of Surgeons verified Level-1-adult and Level-1-pediatric trauma center, which facilitates care for approximately 2500 patients per year. About 20 % of these patients have an Injury Severity Score (ISS) >15. During the inclusion period, the most common type of IVC filter being used at Mayo Clinic was the Günther Tulip IVC filter (Cook Medical Inc, Bloomington, IN). The filter is inserted endovascularly either via the internal jugular vein or the common femoral vein under ultrasound and fluoroscopy guidance. The procedure is often performed by an interventional radiologist. Pharmacological thromboprophylaxis was given to all patients in both groups unless ongoing bleeding or the risk of bleeding was considered high.

### 2.2 Patient inclusion and exclusion

Patients from Mayo Clinic Trauma Registry between 1/2008 and 12/2013 were included. Inclusion criteria were age>15, ISS > 15 upon admission [8], Abbreviated Injury Scale>2 upon admission [8], and survival>24 h after hospital admission. Patients with VTE diagnosed prior to IVC filter placement were excluded. Computational extraction protocols according to the inclusion and exclusion criteria were used to find the patients in the trauma registry database.

### 2.3 End-points and verification

Thromboembolic event data identified in the registry were crosschecked with medical records in order to validate findings. This was done either automatically with computational extraction algorithms (demographics, ICD-9 codes, Current-Procedural-Terminology-Codes) or by free text search of medical records [[9], [10], [11]]. All positive endpoints identified by the trauma registry were confirmed by physician chart review.

PE was diagnosed by computed tomography pulmonary angiography. DVTs in the lower extremities were diagnosed by either duplex ultrasound or computed tomography venography. Referral to diagnostic imaging was based on clinical suspicion.

### 2.4 Mortality verification

All-cause mortality data was ascertained via link to the hospital’s records for death data. Mortality records after hospital discharge was not used in the analysis.

### 2.5 Statistical analysis

Patient characteristics and crude outcomes are presented using the mean (SD) or number (%), as appropriate. Groups were compared using the *t*-test or chi-squared test.

The association of IVC filter placement with in-hospital PE, DVT and mortality was analyzed using a Cox proportional hazards regression model. IVC filter status was entered as an independent variable in the model.

Landmark analysis at a prespecified time point of day 3 after injury was used to adjust for immortal time bias. Group allocation was defined by IVC filter insertion status at day 3 or earlier, and outcomes (PE, DVT, mortality) were only considered if occurring between the landmark and dismissal. In the landmark method, only patients that are still alive or not discharged from the hospital at the landmark time are included in the analysis. Patients discharged from the hospital or dead before or at the landmark were excluded from the analysis. The patients were divided into two categories according to whether they had received an intervention (IVC filter) up to that time, and all interventions after the landmark time were ignored [12]. A sensitivity analysis was performed with different landmarks (day 2, day 3, day 5 and day 7 after injury) and with adjustment for pharmacological thromboprophylaxis.

Differences between IVC filter and non-IVC filter groups were controlled for using a propensity score that was based on variables collected at the time of the initial admission. Patient characteristics at the time of injury included in the propensity score were: age, sex, race, ISS [8], Glasgow Coma Scale (GCS) [8], Abbreviated Injury Scale for head/chest/abdomen/long bones/pelvis/spine [8], transfer between hospitals, Charlson Comorbidity Index [13,14], pulse rate, systolic blood pressure and oxygen saturation. Multiple imputation was used to fill in missing data on baseline characteristics for pulse rate (2% missing values), systolic blood pressure (2%), oxygen saturation (9%) and GCS (5%).

A sensitivity analysis where person-time before IVC filter placement was excluded was also presented to illustrate that this type of analysis may be more prone to bias. All analyses were made with/without adjustment for propensity score and pharmacological thromboprophylaxis. Stata® 15.1 (StataCorp, College Station, TX, USA) was used for statistical analysis.

## 3 Results

### 3.1 Study population and interventions

Of the 1451 consecutive patients reviewed, 282 had IVC filters and 1169 had no IVC filter placed. Baseline patient characteristics of the study population are presented in Table 1. There was a male predominance in both cohorts (67 % vs. 66 %). Mean age was lower in the IVC filter cohort (45.9 vs. 56.9 years). Mean ISS was higher in the IVC filter cohort (29.8 vs. 22.6).

**Table 1:** Baseline patient characteristics, mean (SD), unless specified otherwise.

Characteristics	Overall (n = 1451)	Non-IVC filter (n = 1169)	IVC filter (n = 282)	P-value
Age	54.7 (23.6)	56.9 (23.7)	45.9 (21.4)	<0.001
Male sex, number (%)	955 (66)	767 (66)	188 (67)	0.74
White race, number (%)	1306 (90)	1069 (91)	237 (84)	<0.001
Injury severity score	24 (8.1)	22.6 (6.7)	29.8 (10.6)	<0.001
Glasgow Coma Scale	11.9 (4.8)	12.5 (4.5)	9.7 (5.6)	<0.001
Head AIS	3 (1.8)	3.1 (1.8)	2.5 (1.7)	<0.001
Chest AIS	1.2 (1.5)	1 (1.3)	2.3 (1.4)	<0.001
Abdomen AIS	0.7 (1.4)	0.6 (1.3)	1.3 (1.5)	<0.001
Long bones/pelvis AIS	0.9 (1.4)	0.7 (1.2)	1.9 (1.5)	<0.001
Spine AIS	1.1 (1.4)	0.9 (1.4)	1.9 (1.5)	<0.001
Transferred between hospitals, number (%)	855 (59)	706 (60)	149 (53)	0.02
Charlson Comorbidity Index	1.6 (2.6)	1.7 (2.7)	1.2 (2.0)	0.02
Pulse rate, min^−1^	88.2 (21.7)	85.6 (19.6)	99 (26.1)	<0.001
Systolic blood pressure, mmHg	132.6 (28.0)	135.7 (26.9)	119.4 (29.0)	<0.001
Oxygen saturation (%)	97.3 (4.1)	97.4 (3.5)	97.0 (5.8)	0.16

IVC filter = Inferior Vena Cava filter.

AIS = Abbreviated Injury Scale.

Pharmacological thromboprophylaxis during hospitalization was given more often in the IVC filter cohort (69 % vs. 37 %, p < 0.001). The most common pharmacological thromboprophylaxis being used were low-molecular-weight-heparin (Dalteparin, Enoxaparin) and unfractionated heparin.

In patients where an IVC filter was placed, the insertion rate was 72 % at day 2 or earlier and 84 % at day 3 or earlier. Median time from injury to IVC filter placement was 2 days (range, 0–21 days). Median time from injury to VTE (combined PE and DVT) was 7 days (range, 0–34 days) in the non-IVC filter cohort and 11 days (range, 5–59 days) in the IVC filter cohort. 146 patients (52 %) had a known IVC filter retrieval date, but only 4 was retrieved during the current hospital stay. The median time after hospital discharge when an IVC filter was removed was 98.5 days (range, 3–1000 days). The median length of hospital stay was 6 days (range, 0–128 days) for the non-IVC filter cohort and 15 days (2–148 days) for the IVC filter cohort.

### 3.2 Venous thromboembolism events and deaths

The crude number of PE was 9 (0.8 %) in the non-IVC filter vs. 5 (1.8 %) in the IVC filter cohort (Table 2). For DVT the number was 16 (1.4 %) in the non-IVC filter vs. 17 (6%) in the IVC filter cohort. The number of deaths was 92 (7.9 %) in the non-IVC filter vs. 22 (7.8 %) in the IVC filter cohort. The median time from injury to death was 4.5 days (range, 1–21 days) in the non-IVC filter cohort and 7 days (range, 2–16 days) in the IVC filter cohort.

**Table 2:** In-hospital outcomes, crude numbers (%).

	Non-IVC filter (n = 1169)	IVC filter (n = 282)
Pulmonary Embolism	9 (0.8)	5 (1.8)
Deep Venous Thrombosis	16 (1.4)	17 (6.0)
Mortality	92 (7.9)	22 (7.8)

IVC filter = Inferior Vena Cava filter.

### 3.3 IVC filter and hazard of venous thromboembolism

IVC filter placement was not associated with the hazard of PE when adjusted for propensity score at landmark 2 (HR = 0.37; 95 % CI 0.08,1.71; P = 0.20), landmark 3 (HR = 0.46; 95 % CI 0.12,1.70; P = 0.24) and landmark 5 (HR = 1.52; 95 % CI 0.49,4.70; P = 0.47) (Table 3). However, at landmark 7 (HR = 7.39; 95 % CI 2.50,21.83; P < 0.001) and when person-time before IVC filter placement was excluded (HR = 3.53; 95 % CI 1.44,8.64; P = 0.006), IVC filter placement was associated with the hazard of PE.

**Table 3:** Pulmonary embolism (PE): analysis from landmark to PE or discharge. Dead or discharged at/before landmark were excluded.

Model	No. of patients at risk (no. of PE)			Unadjusted			Adjusted **			Adjusted ***		
	IVC filter	Non-IVC filter	Total	Hazard ratio	95 % CI	P	Hazard ratio	95 % CI	P	Hazard ratio	95 % CI	P
All patients*	282 (5)	1169 (9)	1451 (14)	2.18	1.09,4.35	0.03	3.53	1.44,8.64	0.006	2.90	1.24,6.80	0.014
Landmark set at day 2 after injury	203 (2)	1082 (9)	1285 (11)	0.26	0.06,1.08	0.06	0.37	0.08,1.71	0.20	0.36	0.08,1.61	0.18
Landmark set at day 3 after injury	231 (3)	935 (7)	1166 (10)	0.34	0.10,1.12	0.08	0.46	0.12,1.70	0.24	0.44	0.12,1.59	0.21
Landmark set at day 5 after injury	249 (4)	662 (5)	911 (9)	1.17	0.51,2.70	0.71	1.52	0.49,4.70	0.47	1.38	0.47,4.08	0.56
Landmark set at day 7 after injury	239 (5)	471 (3)	710 (8)	3.43	1.44,8.13	0.005	7.39	2.50,21.83	0.000	5.85	2.04,16.80	0.001

95 % CI = 95 % Confidence Interval.

IVC filter = Inferior Vena Cava filter.

* Person-time before IVC filter placement (immortal time) excluded.

** Adjusted for propensity score with variables included as shown in Table 1.

*** Adjusted for propensity score and pharmacological thromboprophylaxis during hospitalization.

IVC filter placement was associated with the hazard of DVT when adjusted for propensity score at landmark 2 (HR= 2.34; 95 % CI 1.10,5.00; P = 0.03), landmark 3 (HR = 2.73; 95 % CI 1.28,5.85; P = 0.01), landmark 7 (HR = 5.48; 95 %CI 2.34,12.83; P < 0.001) and when person-time before IVC filter placement was excluded (HR = 3.91; 1.84,8.30; P < 0.001) (Table 4). At landmark 5, the increased hazard of DVT with IVC filter in place was not statistically significant (HR = 2.13; 95 % CI 0.92,4.89; P = 0.08).

**Table 4:** Deep venous thrombosis (DVT): analysis from landmark to DVT or discharge. Dead or discharged at/before landmark were excluded.

Model	No. of patients at risk (no. of DVT)			Unadjusted			Adjusted **			Adjusted ***		
	IVC filter	Non-IVC filter	Total	Hazard ratio	95 % CI	P	Hazard ratio	95 % CI	P	Hazard ratio	95 % CI	P
All patients*	282 (17)	1169 (16)	1451 (33)	2.79	1.52,5.12	0.001	3.91	1.84,8.30	0.000	3.38	1.63,7.01	0.001
Landmark set at day 2 after injury	203 (13)	1082 (18)	1285 (31)	1.77	0.91,3.42	0.09	2.34	1.10,5.00	0.03	2.19	1.04,4.61	0.04
Landmark set at day 3 after injury	231 (15)	935 (16)	1166 (31)	1.83	0.97,3.47	0.06	2.73	1.28,5.85	0.01	2.51	1.19,5.27	0.02
Landmark set at day 5 after injury	249 (15)	662 (14)	911 (29)	1.36	0.71,2.60	0.36	2.13	0.92,4.89	0.08	2.01	0.89,4.51	0.09
Landmark set at day 7 after injury	239 (14)	471 (9)	710 (23)	2.59	1.29,5.19	0.10	5.48	2.34,12.83	0.000	4.69	2.05,10.76	<0.001

95 % CI = 95 % Confidence Interval.

IVC filter = Inferior Vena Cava filter.

* Person-time before IVC filter placement (immortal time) excluded.

** Adjusted for propensity score with variables included as shown in Table 1.

*** Adjusted for propensity score and pharmacological thromboprophylaxis during hospitalization.

### 3.4 IVC filter and hazard of death

IVC filter placement was not associated with all-cause mortality at landmark 2 (HR = 1.16; 95 % CI 0.71,1.88; P = 0.56), landmark 3 (HR = 1.02; 95 % CI 0.60,1.75; P = 0.93), landmark 5 (HR = 0.76; 95 % CI 0.40,1.47; P = 0.42) and landmark 7 (HR = 0.82; 95 % CI 0.38,1.74; P = 0.60) (Table 5). When person-time before IVC filter placement was excluded, IVC filter placement was associated with the hazard of all-cause mortality (HR = 0.50; 95 % CI 0.33,0.78; P < 0.002).

**Table 5:** Mortality: analysis from landmark to dead or discharge. Dead or discharged at/before landmark were excluded.

Model	No. of patients at risk (no. of deaths)			Unadjusted			Adjusted **			Adjusted ***		
	IVC filter	Non-IVC filter	Total	Hazard ratio	95 % CI	P	Hazard ratio	95 % CI	P	Hazard ratio	95 % CI	P
All patients*	282 (22)	1169 (92)	1451 (114)	0.89	0.62,1.27	0.51	0.50	0.33,0.78	0.002	0.51	0.33,0.80	0.004
Landmark set at day 2 after injury	203 (15)	1082 (70)	1285 (85)	1.43	0.95,2.15	0.09	1.16	0.71,1.88	0.56	1.16	0.71,1.91	0.56
Landmark set at day 3 after injury	231 (15)	935 (59)	1166 (74)	1.17	0.74,1.84	0.50	1.02	0.60,1.75	0.93	1.03	0.60,1.77	0.92
Landmark set at day 5 after injury	249 (13)	662 (37)	911 (50)	0.87	0.51,1.49	0.61	0.76	0.40,1.47	0.42	0.77	0.40,1.49	0.44
Landmark set at day 7 after injury	239 (9)	471 (25)	710 (34)	0.87	0.47,1.62	0.66	0.82	0.38,1.74	0.60	0.83	0.39,1.77	0.63

95 % CI = 95 % Confidence Interval.

IVC filter = Inferior Vena Cava filter.

* Person-time before IVC filter placement (immortal time) excluded.

** Adjusted for propensity score with variables included as shown in Table 1.

*** Adjusted for propensity score and pharmacological thromboprophylaxis during hospitalization.

Additional adjustment for pharmacological thromboprophylaxis did not significantly alter the results for any of the analyses.

## 4 Discussion

The main findings of this retrospective comparative study were that prophylactic IVC filter placement in patients after severe trauma was not associated with the hazard of in-hospital PE or mortality. However, an increased hazard of in-hospital DVT was observed in patients where an IVC filter was inserted.

Use of IVC filters in patients following severe trauma without recent history of VTE is controversial due to conflicting reports of efficacy. Some observational studies have reported a lower incidence of PE or mortality following IVC filter placement while others have not shown this association [[15], [16], [17], [18], [19]]. There are also conflicting reports whether IVC filters may pose an increased risk of VTE and mortality [[19], [20], [21]].

Currently, there are only two randomized controlled trials investigating the role of IVC filters in trauma patients [7,22]. The first trial was a pilot study from 2011 demonstrated that it was feasible to perform randomization comparing prophylactic IVC filters versus no IVC filter for prevention of PE in high-risk trauma patients [22]. The study was, however, underpowered and included only 38 patients. The recent larger trial demonstrated that early prophylactic placement of IVC filters in trauma patients did not lower the risk of PE or mortality at 90 days [7]. The trial randomized 240 severely injured patients (ISS > 15) with contraindication to pharmacological thromboprophylaxis to receive an IVC filter or not within 72 h after admission. Pharmacological thromboprophylaxis was, however, initiated within 7 days after injury in 67 % of the patients enrolled in the study.

A recent meta-analysis which included the two randomized controlled trials, demonstrated that IVC filters after major trauma may reduce symptomatic but not fatal pulmonary embolism [7,22,23]. Major trauma was defined ISS > 15 or any reason to delay initiation of pharmacological thromboprophylaxis.

In a propensity matched controlled study of 451 trauma patients where an IVC filter was inserted matched with 1343 controls without an IVC filter, no difference was found in long-term survival between the groups irrespective of the presence of VTE or not [18].

In contrast, the present study analyzed in-hospital outcomes of PE, DVT and mortality but did not include follow-up time after hospital discharge. Although with different study design and follow-up time, the results of the present study are in line with the previous studies [7,18].

The use of IVC filters in other populations has also failed to demonstrate mortality benefits. In obese patients undergoing bariatric surgery, a systematic review did not show evidence that preoperative IVC filter insertion reduced PE-related mortality in patients with multiple risk factors for VTE [24]. In patients with recent history of VTE, two randomized trials have also failed to show survival benefit of IVC filters [21,25].

A strength of our study is the large number of patients studied from a large trauma center during a time period of six years. With 1451 patients in this study, the sample size is larger than in the randomized trials and may better reflect the real-world practice. Compared to some other retrospective studies on this topic, our analyses address selection bias and immortality bias. To account for this, we used a propensity score, based on general and trauma specific variables collected upon admission, in the Cox regression model in order to balance differences in baseline characteristics between the groups. [26,27] To address immortal time bias, landmark analysis was used and the patients who were dead or discharged at or before the landmark were excluded from the study [28,29].

Another way to deal with immortal time is the Mantel-Byar method, also called time-dependent approach, which is considered the gold standard method for handling immortal person-time bias [6,30]. This method removes bias when patients are compared according to their response status (IVC filter placement) at various periods during follow up. The landmark method is however easier to apply and can be performed in any standard statistical software. On the other hand, the choice of landmark analysis should be done carefully and guided by clinical relevance. In the present study we chose the main landmark close to the median time of IVC filter insertion after injury. Landmarks further away from this would have excluded many patients due to death or hospital discharge and underpowered the analysis.

There are several limitations in our study. Limited number of variables were available for inclusion in the propensity score model. These lacking variables as well as unmeasured variables may result in residual confounding. Obesity is a known risk factor for VTE, but body weight was not available for inclusion in our propensity score model [31,32]. Data from when pharmacological thromboprophylaxis was initiated as well as the duration and the dosage of the therapy was also not available. Additionally, there was no routine surveillance program for VTE during the time-period the study was conducted. Surveillance programs may increase the awareness of VTE and may detect more subclinical DVT but may not necessarily improve clinical outcomes by reducing PE [33]. This study used time to in-hospital events and mortality. This time may be influenced by the timing of hospital discharge, and a standardized follow-up period of 30 or 90 days may have been preferable. However, most of the events would occur during the hospital stay.

Patients with IVC filter placed may indicate that providers or the teams were more vigilant to VTE symptoms and therefore ordered more imaging or surveillance scans, which might increase the number of VTE outcomes in that group. However, adherence to an evidence-based thromboprophylaxis protocol may play a more important role than surveillance in preventing VTE in trauma patients [34]. Additionally, there was no written policy for IVC filter insertion in trauma patients at Mayo Clinic during that time period. The decision for placement of an IVC filter was on a case by case basis.

This study did not show a benefit of prophylactic IVC filter placement in severely injured patients in reducing PE but was associated with increased hazard of DVT. Most importantly, our study did not find an association between IVC filter placement and all-cause mortality.

## 5 Conclusions

Prophylactic IVC filter placement in severe trauma patients was not associated with the hazard of PE or mortality. Nevertheless, an increased hazard of DVT was observed with filter present.

## Ethical approval

This study was approved by the Institutional Review Board at Mayo Clinic in accordance with the local regulations governing clinical research.

The large number of patients needed for this study precluded informed consent.

## Funding

This study was not supported by any funding.

## Declaration of Competing Interest

The authors declare that they have no conflict of interest
